# Targeting myeloid-derived suppressor cells in the tumor microenvironment: potential therapeutic approaches for osteosarcoma

**DOI:** 10.32604/or.2024.056860

**Published:** 2025-02-28

**Authors:** HYE IN KA, SE HWAN MUN, SORA HAN, YOUNG YANG

**Affiliations:** Research Institute of Women’s Health, Sookmyung Women’s University, Seoul, 04312, Republic of Korea

**Keywords:** Immune cells, Tumor microenvironment (TME), Bone malignancy, Myeloid-derived suppressor cells (MDSC)

## Abstract

Osteosarcoma is a bone malignancy characterized by strong invasiveness and rapid disease progression. The tumor microenvironment of osteosarcoma contains various types of immune cells, including myeloid-derived suppressor cells, macrophages, T cells, and B cells. Imbalances of these immune cells can promote the proliferation and metastasis of osteosarcoma. Recent studies have indicated a substantial increase in the levels of myeloid-derived suppressor cells, an immune cell associated with immunosuppressive and pro-cancer effects, in the peripheral blood of patients with osteosarcoma. Moreover, the levels of the pro-inflammatory cytokine interleukin 18 are positively correlated with those of myeloid-derived suppressor cells in the peripheral blood of animal models of osteosarcoma. In this review, we explore the function of myeloid-derived suppressor cells in osteosarcoma based on the clinical diagnoses of patients with osteosarcoma and discuss future therapeutic approaches for targeting osteosarcoma. Targeting myeloid-derived suppressor cells represents a promising approach to improving the prognosis and survival rates of patients with osteosarcoma.

## Introduction

### Myeloid-derived suppressor cells in osteosarcoma

Myeloid-derived suppressor cells (MDSCs) are classified based on the presence of cytoplasmic granules into two types: granulocytic/polymorphonuclear MDSCs (PMN-MDSCs) and monocytic MDSCs (M-MDSCs) [1]. PMN-MDSCs, the largest proportion of immune cells in all types of cancer, resemble neutrophils, whereas M-MDSCs are similar to monocytes. In mice, MDSCs are identified based on the expression of the markers CD11b and Gr1. PMN-MDSCs show high Ly6G and low Ly6C levels, whereas M-MDSCs express low Ly6G and high Ly6C levels [2–4]. In humans, MDSCs are distinguished based on the expression of markers such as CD33, CD14, CD15, and low HLA-DR levels [4]. Specifically, human PMN-MDSCs are identified as Lin^−^HLA^-^DR^−/low^CD33^+^ or Lin^−^HLA^-^DR^−/low^CD11b^+^CD14^−^CD15^+^CD33^+^, whereas human M-MDSCs are characterized as CD14^+^HLA^-^DR^−/low^ or Lin^−^HLA^-^DR^−/low^CD11b^+^CD14^+^CD15^−^ [3]. A newly identified type, early-stage MDSCs, expresses both granulocytic and monocytic markers [5]. MDSCs are a diverse group of immature myeloid cells that accumulate during cancer proliferation and other pathological conditions [6,7]. They are potent immune suppressors and facilitate tumor growth and metastasis through various mechanisms, such as suppression of T-cell activation and promotion of tumor immune evasion in various cancers including melanoma, non-small cell lung cancer, and osteosarcoma [8]. Conversely, MDSCs perform distinct functions in autoimmune diseases such as rheumatoid arthritis [9–12] having protective pro-inflammatory effects. Thus, dual mechanisms and functions of MDSCs have been identified across different disease contexts.

Osteosarcoma is a highly aggressive bone malignancy characterized by rapid progression and a high potential for metastasis, particularly affecting children, adolescents, and young adults [13]. The tumor microenvironment (TME) in osteosarcoma is a complex and dynamic network comprising cancer cells along with various stromal and immune cells, including MDSCs, macrophages, T cells, and B cells [14]. These components interact to create an environment that supports tumor growth, immune evasion, and metastasis. An imbalance among these immune cells can lead to an immunosuppressive TME which favors tumor progression and therapeutic resistance. Recent research has highlighted the important role of MDSCs in the TME of osteosarcoma, contributing to immunosuppression and tumor progression [15]. The number of MDSCs is significantly increased in patients with osteosarcoma, indicating their critical role in the TME of osteosarcoma. A review of MDSCs in musculoskeletal diseases reported that MDSCs, particularly PMN-MDSCs, accumulate in the TME and inhibit T cell-mediated immune responses, induced by high expression levels of interleukin (IL)-18 and C-X-C motif chemokine ligand 12 [16]. Blocking these factors improved the efficacy of anti-programmed cell death protein 1 (PD-1) treatment in mice. Using an osteosarcoma tumor model, Guan et al. found that the levels of MDSCs are positively correlated with those of IL-18, suggesting that IL-18 may attract MDSCs into the tumor parenchyma. A combination therapy with IL-18 inhibition and anti-PD-1 decreased tumor burden and increased T-cell infiltration [17]. Long et al. reported that osteosarcoma-induced MDSCs are associated with poor chimeric antigen receptor (CAR) T-cell efficacy against xenografts [18]. Osteosarcomas significantly induced the proliferation of CD11b^+^Ly6G^+^ and CD11b^+^Ly6C^+^ myeloid cell populations compared to nontumor-bearing controls in a murine model. Clinically, proliferating monocytic and granulocytic MDSCs adversely affected the efficiency of GD2-CAR T cells generated from apheresis products of patients with osteosarcoma [19].

### The multifaceted immunosuppressive roles of MDSCs in the TME of osteosarcoma: Crosstalk with other immune cells

Osteosarcoma tissue forms a heterogeneous and complex immune microenvironment with extensive and varied immune cell infiltration [20]. This TME accelerates cancer cell growth within the bone by establishing an immunosuppressive milieu that supports cancer cell survival and proliferation [20,21]. Guan et al. reported significant increases in MDSC populations in the peripheral blood of patients with osteosarcoma [17]. These cells heavily infiltrate the osteosarcoma TME, promoting cancer progression by inhibiting the proliferation of anti-cancer cytotoxic T cells (CTLs) and preventing CTL-associated anti-PD-1 cancer therapy [22]. By interacting with various immune cells, including T cells, dendritic cells (DCs), and natural killer (NK) cells, MDSCs create an immunosuppressive environment. They also modulate immunosuppressive functions through humoral interactions with osteoblasts, osteoclasts, chondrocytes, and other stromal cells in the osteosarcoma TME [20,23]. Additionally, MDSCs downregulate the anti-cancer immune response by further differentiating into cancer-favoring and immunosuppressive DCs, tumor-associated macrophages (TAMs), and tumor-associated neutrophils [20,24]. However, this review focuses on how MDSCs contribute to the immunosuppressive TME by interacting with immune cells rather than their differentiation into other immunosuppressive cell types.

T cell-targeted immunotherapy is a promising strategy for treating osteosarcoma [17,25–27]. Considering that MDSCs are potent inhibitors of T cell-mediated anti-cancer immunity, understanding how MDSCs dysregulate T cells is crucial for optimizing T cell-mediated anti-osteosarcoma therapies. MDSCs interact closely with T cells, suppressing their antitumor activity through humoral mechanisms and direct cell–cell interactions. MDSCs express and secrete high levels of arginase 1 (ARG1) into the TME, thus depleting L-arginine, an essential amino acid for T-cell metabolism [28]. This depletion leads to T-cell cycle arrest before entering the G1 phase by suppressing cyclin D3 expression [28]. MDSC-derived ARG1 also downregulates the CD3ζ transmembrane component of the T-cell receptor (TCR), thereby disrupting T-cell activation via TCR engagement [28]. Additionally, MDSCs deplete cysteine, another critical amino acid for T-cell proliferation and activation. Cysteine is the rate-limiting substrate for the synthesis of the tripeptide glutathione [29]. T cells acquire cysteine by interacting with antigen-presenting cells via a neutral amino acid transporter. MDSCs compete with antigen-presenting cells, including macrophages and DCs, thereby interrupting the cysteine uptake by T cells [28,30]. Furthermore, MDSC-derived indoleamine 2,3-dioxygenase (IDO) depletes tryptophan, leading to T-cell cycle arrest in the mid-G1 phase and inhibition of T-cell proliferation [28,31].

Reactive oxygen species (ROS) and nitric oxide (NO), major humoral factors produced by MDSCs, disrupt T-cell functions. PMN-MDSCs primarily produce ROS, whereas M-MDSCs are characterized by enhanced NO production. TME-derived IL-6 activates the Janus kinase 2 (JAK)/signal transducer and activator of the transcription 3 (STAT3) signaling pathways, thereby increasing the expression of nicotinamide adenine dinucleotide phosphate hydrogen oxidase 2 (NOX2), which mediates ROS production in PMN-MDSCs [32,33]. M-MDSCs express high levels of inducible nitric oxide synthase (iNOS), leading to significantly increased NO production. Hypoxic conditions within solid tumors also upregulate iNOS and ARG1 expression in MDSCs [34]. ROS and NO produce the byproduct peroxynitrite, which disrupts the conjugation of the TCR component CD8 with major histocompatibility complex molecules on cancer cell surfaces, thereby reducing the cytotoxic activity of CD8^+^ CTLs [28,35]. Additionally, MDSCs inactivate the T cell-activating IL-2 signaling pathway and destabilize IL-2 mRNA, thus decreasing IL-2 production and secretion, which suppresses T-cell activity [36,37]. This multifaceted suppression orchestrated by MDSC-derived ROS and NO underscores the critical role of MDSCs in modulating anti-cancer responses within the TME.

Programmed death ligand 1 (PD-L1), expressed on the surfaces of PMN-MDSCs and M-MDSCs directly binds PD-1 on CD8^+^ T cells. This interaction inhibits T-cell functions through the PD-1–PD-L1 signaling pathway. The hypoxic conditions within the TME increase PD-L1 expression in infiltrating MDSCs by enhancing the expression and stability of hypoxia-inducible factor 1α, which promotes PD-L1 transcription in MDSCs [38,39]. Anti-PD-1/anti-PD-L1 therapy is a promising strategy for treating various types of cancers, as cancer cells express high levels of PD-L1 to evade anti-cancer T-cell activity [40,41]. However, patients with osteosarcoma are resistant to PD-1/PD-L1 inhibitors, and anti-PD-1 monotherapy has been ineffective against osteosarcoma in a murine model of K7M2 osteosarcoma grafts [22]. Interestingly, these authors found that combining a C-X-C chemokine receptor (CXCR)4 antagonist with an anti-PD-1 monoclonal antibody can overcome the limitations of anti-PD-1 therapy in their osteosarcoma model. They reported that continuous substantial MDSC infiltration into the TME undermines the efficacy of anti-PD-1 therapy. The CXCR4 antagonist reduced MDSC chemotaxis into the osteosarcoma TME, thereby enhancing the antitumor effects of anti-PD-1 therapy [22]. However, the impact of MDSC suppression, the identification of effective strategies to inhibit MDSC infiltration into the tumor site, and the implications for anti-osteosarcoma therapy need further preclinical and clinical evaluation.

Although the major immunosuppressive and anti-cancer strategies of MDSCs are primarily mediated through their crosstalk with T cells, MDSCs also interact with other immune cells, such as NK cells, by altering the expression levels of natural cytotoxicity receptors on the surfaces of NK cells. For instance, MDSCs downregulate CD247 [42], natural cytotoxicity triggering receptor 3 [43], and natural killer group 2D, impairing the anti-cancer function of interferon-gamma production by NK cells [44]. The role of MDSC–NK cell crosstalk in the TME of osteosarcoma remains largely unknown, particularly regarding the pathophysiology and metastasis of osteosarcoma, highlighting the need for further investigation. These MDSC interactions in osteosarcoma collectively create a complex immunosuppressive and oncogenic network within the TME, effectively inhibiting multiple arms of the immune system, including both innate (NK cells, DCs) and adaptive (T cells) responses.

MDSCs can interact not only with immune cells but also directly with cancer cells within the osteosarcoma TME. Taylor et al. demonstrated that MDSCs expressing high levels of IL-1β bind directly to osteosarcoma cells through their interaction with the IL1R1 receptor, which is highly expressed in osteosarcoma. This binding activates downstream nuclear factor kappa-light-chain-enhancer of activated B cells (NF-κB) signaling [45]. NF-κB is recognized as a critical oncogene in osteosarcoma, promoting cell proliferation, survival, migration, and invasion, while inhibiting apoptosis in 143B and MG63 human osteosarcoma cells [46].

In summary, MDSCs play crucial roles in shaping the immunosuppressive TME of osteosarcoma. They suppress T-cell functions, promote the expansion of regulatory T cells, reduce NK cell-mediated antitumor immunity, and impair DC-mediated antigen presentation. By interacting with macrophages and differentiating into immunosuppressive cell types, MDSCs further reinforce the immunosuppressive network within the TME. Understanding these multifaceted interactions and mechanisms is essential for developing effective therapeutic strategies to counteract MDSC-mediated immunosuppression and enhance antitumor immune responses in osteosarcoma.

### The role of MDSCs in osteosarcoma metastasis

MDSCs also play a considerable role in promoting osteosarcoma metastasis through several mechanisms. They contribute to forming a pre-metastatic niche, creating a favorable environment for tumor cells to establish metastases by secreting factors that alter the extracellular matrix, promoting angiogenesis, and recruiting other immunosuppressive cells [47]. MDSCs also enhance tumor cell invasion by producing factors that facilitate the spread of osteosarcoma cells to distant sites [47]. They create an immunosuppressive microenvironment that allows tumor cells to evade immune detection and destruction by suppressing T-cell function through ROS production, depleting L-arginine, inhibiting NK-cell activity through phagocytosis, and promoting Treg proliferation [33]. Tumors recruit MDSCs through various chemokine pathways, including C-C motif chemokine ligand (CCL)2/CCL12–C-C chemokine receptor (CCR)2, CCL3/4/5–CCR5, and CXCL8–CXCR1/CXCR2, which subsequently promote metastasis through similar chemokine axes [33]. These MDSCs also secrete pro-metastatic factors such as transforming growth factor beta, S100A8/A9, vascular endothelial growth factor (VEGF), and exosomes, which interact with various cells in the TME, making it more conducive to metastasis [8,33,48]. Additionally, MDSCs enhance metastasis by promoting β-adrenergic signaling and the IL-6–STAT3 pathway and can differentiate into osteoclasts in bone metastatic cancers, thereby contributing to bone destruction and promoting tumor growth [49]. In bone metastatic cancers, MDSC-derived NO mediates immunosuppression and promotes osteoclastogenesis, further facilitating metastasis. Moreover, the presence of MDSCs in the TME can impair the efficacy of immunotherapeutic drugs, such as PD-1 inhibitors, by suppressing T-cell responses [50].

## Targeting MDSCs in the TME: Potential Approaches for Osteosarcoma Therapy

**Figure 1:**
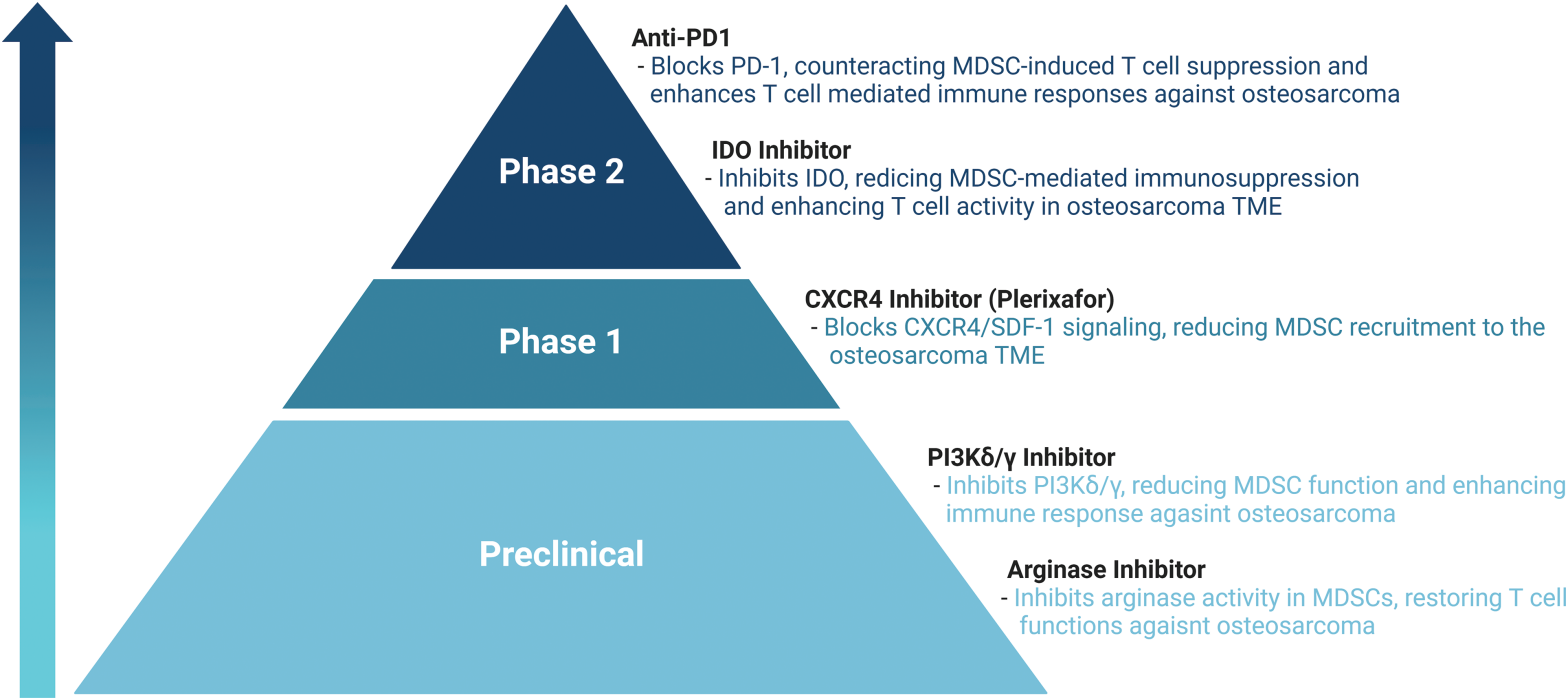
Therapeutic approaches targeting MDSCs in TME.

To improve the survival rates of patients with osteosarcoma, various treatment approaches are employed, including surgery [51,52], chemotherapy [53], immunotherapy [54,55], and targeted therapy [56–59]. Each method plays a crucial role in the comprehensive management of the disease, aiming to enhance patient outcomes and increase the likelihood of long-term survival. Recent research has focused on targeting MDSCs within the TME as a promising therapeutic strategy for cancer, including osteosarcoma (Table A1, Fig. 1). However, research on cancer therapies targeting MDSCs specifically in osteosarcoma is limited. Therefore, we aim to describe tumor therapies that target MDSCs in the TME and discuss the potential application of these approaches in the treatment of osteosarcoma.

### Targeting MDSC inhibition

Blocking the activation of MDSCs is a critical therapeutic strategy to mitigate their immunosuppressive effects in the TME. This approach involves targeting key signaling pathways and transcription factors essential for MDSC activation. Several signaling pathways, including the STAT3, CCAAT-enhancer-binding protein (C/EBP) β, and interferon regulatory factor 8 (IRF8) pathways, are linked to MDSC proliferation and activation. Inhibiting the STAT3 pathway can reduce MDSC proliferation and their immunosuppressive activities [60,61]. STAT3 downregulation, caused by activated CD45 phosphatase, has been observed in M-MDSCs, leading to a unique differentiation into TAMs [62]. Preclinically, STAT3 inhibitors have shown promising results by reducing MDSC numbers and enhancing the efficacy of other cancer therapies. Inhibitors of the JAK–STAT3 pathway, such as JSI-124, significantly reduce MDSC numbers and suppress their activity, leading to enhanced antitumor immune responses [63]. STAT3 plays major roles in the progression and metastasis of osteosarcoma. Inhibition of STAT3 blocks protein synthesis and reduces tumor metastasis in osteosarcoma cells. This has led to preclinical studies exploring STAT3 inhibitors as a potential therapeutic approach in osteosarcoma [64,65]. The C/EBPβ pathway is regulated by IL-6 and controls the expression of immune-suppressive genes (such as those encoding ARG1, iNOS, and NOX2), thereby regulating the differentiation and function of MDSCs. C/EBPβ promotes MDSC generation in the bone marrow or spleen and is closely associated with the expression of granulocyte-macrophage colony-stimulating factor (GM-CSF) and granulocyte colony-stimulating factor (G-CSF) in myeloid cells. Targeting C/EBPβ in osteosarcoma treatment is a less common strategy. However, it has been reported that M-MDSCs are reduced in the spleen of C/EBPβ-deficient mice [66–68], and this transcription factor is known to regulate immunosuppressive genes and participate in the TME in various cancers [69]. Therefore, there is potential for its application in combination therapy for osteosarcoma. IRF8 pathways are also involved in MDSC differentiation and function. Downregulation of IRF8 induces the production of PMN-MDSCs. *In vivo*, IRF8 overexpression inhibits MDSC proliferation and enhances antitumor efficacy through the STAT3 and STAT5 signaling pathways. IRF8 overexpression selectively induces the expansion of granulocytes and PMN-MDSCs while not significantly affecting monocytes or M-MDSCs. Other downstream effectors in MDSC activation include S100A9 and NOX2, which are involved in ROS production. Inhibitors targeting these molecules can effectively impair MDSC function and enhance antitumor immune responses [70,71]. Research directly targeting IRF8 in osteosarcoma is not well established. However, its role in modulating immune responses suggests it might be a future target for therapies aimed at enhancing antitumor immunity in patients with osteosarcoma [65]. A study by Li et al. demonstrated that disruption of the COX-2/PGE2 pathway and the use of phosphodiesterase (PDE) 5 inhibitors, such as sildenafil, vardenafil, and tadalafil, neutralize MDSC immunosuppressive capacities [7]. Sildenafil reduces ARG1 and iNOS expression in MDSCs, thereby inhibiting their immunosuppressive functions [72]. Furthermore, sildenafil prolongs the survival of melanoma-bearing mice by reducing MDSC levels and activity, leading to restored CD8^+^ T-cell infiltration and function in the TME [73]. PDE inhibitors can reactivate antitumor immunity by enhancing T- and NK-cell functions. In preclinical murine models, the administration of sildenafil and tadalafil significantly reduces MDSC levels and their suppressive activity, leading to improved T-cell proliferation and tumor cell apoptosis. These agents not only destroy tumor cells but also diminish the suppressive effects of MDSCs on the immune system, thereby restoring the antitumor immune response. Several studies focus on the pleiotropic effects of PDE5 inhibitors, which include reducing tumor growth by enhancing NO signaling and inhibiting tumor angiogenesis. These mechanisms are being explored in preclinical models of osteosarcoma. For instance, the results of some studies suggest that by modulating blood flow and reducing hypoxia, PDE5 inhibitors may reduce the aggressive spread of cancer cells and improve the efficacy of other treatments like chemotherapy [74,75]. This potential can also be seen in research on osteosarcoma using the phosphodiesterase family member PDE2. In a study investigating the role of PDE2 in human osteosarcoma cells, PDE2 inhibition regulated cell proliferation and migration. This suggests potential therapeutic implications for targeting PDE pathways in osteosarcoma, particularly in manipulating cAMP and cGMP signaling in osteosarcoma cells [76].

### Targeting MDSC recruitment

MDSCs are recruited to the TME through various tumor-derived growth factors such as GM-CSF, G-CSF, VEGF, macrophage colony-stimulating factor (M-CSF), and IL-6. Targeting these pathways can reduce MDSC recruitment. GM-CSF drives the proliferation and immunosuppressive activity of MDSCs. Inhibiting GM-CSF can reduce MDSC proliferation and subdue their immunosuppressive functions [77]. G-CSF promotes MDSC proliferation, and targeting G-CSF can help limit the accumulation of these cells in the TME [78]. VEGF is involved in MDSC recruitment and impairs DC maturation, thereby promoting an immunosuppressive TME. Anti-VEGF therapies can reduce MDSC levels and improve antitumor immune responses [79]. IL-6 positively correlates with MDSC levels in various cancers. Targeting IL-6 can disrupt MDSC recruitment and support the anti-cancer ability of the immune system [80]. GM-CSF, G-CSF, VEGF, M-CSF, and IL-6 are all key cytokines that can be linked to osteosarcoma therapy due to their roles in immune regulation, inflammation, and tumor growth. GM-CSF and G-CSF have been used to stimulate immune responses, whereas VEGF is often targeted to inhibit tumor angiogenesis. M-CSF and IL-6 also play roles in promoting macrophage activity and inflammation, which can influence tumor progression. Emerging therapies explore how modulating these cytokines can enhance treatment outcomes in osteosarcoma [81–84].

According to chemokine studies examining MDSCs, the CCL2–CCR2 axis is crucial for recruiting MDSCs to tumor sites. Inhibiting this axis can reduce MDSC accumulation in the TME, thereby alleviating immunosuppression. Blocking CCR2 reduces MDSC migration to tumors and enhances antitumor T-cell responses. This strategy helps in reprogramming the TME to support antitumor immunity [85,86]. CXCR2 plays a considerable role in the trafficking of PMN-MDSCs. Inhibiting CXCR2 disrupts the recruitment of these cells to the tumor site, thereby reducing their immunosuppressive effects. CXCR2 inhibitors, such as SX-682, impair PMN-MDSC trafficking, which subsequently enhances the efficacy of T cell- and NK cell-based immunotherapies by allowing better immune cell infiltration and activity within the tumor [85,87]. In osteosarcoma, crucial roles of CCL2 and its receptor CCR2 have been identified. The CCL2–CCR2 pathway contributes to the migration of immune cells, such as TAMs, as well as to tumor growth and metastasis within the osteosarcoma microenvironment. Study findings suggest that inhibition of the CCL2–CCR2 pathway can suppress the progression of osteosarcoma and prevent bone destruction [88–90]. Other studies have demonstrated that the SDF-1–CXCR4 signaling axis plays a crucial role in the accumulation of MDSCs within the TME. Notably, the CXCR4 antagonist AMD3100 showed a synergistic effect when used in combination with anti-PD-1 antibodies in murine models of osteosarcoma. These findings suggest that co-administration of CXCR4 antagonists with PD-1 inhibitors might be a promising therapeutic strategy for improving treatment outcomes in patients with osteosarcoma [22]. This research highlights that inhibiting the SDF-1-CXCR4 axis, a key signaling pathway that regulates the accumulation and function of immune cells in osteosarcoma, can suppress tumor growth and enhance immune responses. In particular, the combination of CXCR4 antagonists with immune checkpoint inhibitors (ICIs, such as anti-PD-1 drugs) has the potential to maximize therapeutic efficacy [91].

### Targeting MDSC reprogramming

Reprogramming MDSCs within the TME aims to transform these immunosuppressive cells into non-suppressive or even antitumor immune cells. This approach seeks to enhance the body’s natural immune response against cancer by modifying the behavior and function of MDSCs [92]. One strategy involves inducing the differentiation of MDSCs into mature DCs or macrophages that do not support tumor growth. This differentiation can be promoted using cytokines or specific drugs. All-trans retinoic acid (ATRA) supports antitumor therapy by reprogramming MDSCs within the TME [93,94]. Tobin et al. reported that ATRA reduces the expression of immunosuppressive genes, including those encoding PD-L1, IL-10, and IDO, in MDSCs [95]. The use of ATRA promotes the differentiation of MDSCs into mature cells, thereby diminishing their suppressive activity [96–98]. In an osteosarcoma study, ATRA decreased osteosarcoma metastases by reprogramming MDSCs in the TME. This provides insights into how such strategies can convert immune-resistant “cold” tumors into “hot” tumors, making them more responsive to immunotherapies [99].

### Targeting MDSC metabolic pathways

Targeting the metabolic pathways of MDSCs is a promising therapeutic strategy to overcome immunosuppressive TME. These cells express high levels of ARG1, which depletes arginine, and consequently inhibit T-cell proliferation and function. Inhibitors of ARG1 can restore T-cell activity and enhance antitumor immunity [100–102]. Additionally, MDSCs produce NO through iNOS, thereby suppressing T-cell function and promoting tumor growth. iNOS competes with ARG1 for the same substrate and metabolizes L-arginine into citrulline and NO in tumor progression and T-cell activation [103]. Therefore, iNOS inhibitors may reduce MDSC-mediated immunosuppression and improve the effectiveness of other cancer therapies [104–106]. Targeting ARG1 and iNOS involved in the L-arginine pathway that can induce the inhibition of L-arginine metabolism. It is an essential strategy to activate T-cell function and decrease the activity of immune-suppressive cells. Rodriguez et al. found that L-arginine starvation arrests the cell cycle from the G1 to the S phase with impairment of cyclin D expression [102]. The suppressive effects of MDSCs are attributed to L-arginine metabolism. The Warburg effect is a hallmark of cancer characterized by metabolic reprogramming in tumors [107]. Excess lactate is transported across membranes by the monocarboxylate transporters (MCT) 1 and MCT4, which are crucial for tumor aggressiveness [108,109]. Lymphoid-derived myeloid-derived suppressor cells often upregulate glycolytic pathways to meet their energy demands. Inhibiting key glycolytic enzymes such as hexokinase 2 (HK2) or pyruvate kinase M2 (PKM2) can reduce MDSC survival and function [110–112]. Targeting these pathways may shift the TME toward a more immune-permissive state. HK2 and PKM2 impair glucose metabolism, thereby affecting the survival and function of both MDSCs and tumor cells. This intervention disrupts glycolysis, which is crucial for the energy supply and function of both MDSCs and tumor cells, potentially enhancing antitumor immunity [113,114]. Thus, glycolytic inhibition could be a viable strategy to modulate the immune environment in favor of antitumor responses, making the TME less supportive of cancer progression. Lactate is an immunosuppressive metabolite that promotes tumor expansion, induces angiogenesis, stimulates amino acid metabolism, inhibits CTLs, NK cells, and DCs, and further impedes the antitumor response [109]. Additionally, lactate promotes cancer growth by inducing protumor abilities, such as MDSC differentiation [115]. Lactate-induced MDSCs directly inhibit the antitumor response of immune cells. Moreover, the levels of immunosuppressive MDSCs increase when cultured with high concentrations of lactate [116]. Research on osteosarcoma therapies targeting L-arginine, lactate, and HK2 primarily aims to inhibit cancer cell metabolism and enhance immune responses. Therapeutic strategies that target the glycolytic pathway can be pivotal in promoting antitumor immune responses and inhibiting tumor cell growth. For instance, using HK2 inhibitors can block glycolysis in cancer cells, disrupting their energy production. Additionally, by reducing lactate production, these treatments can help improve the immunosuppressive environment, thereby enhancing immune function and limiting tumor progression [117]. Moreover, MDSCs exhibit altered lipid metabolism, characterized by increased fatty acid uptake and oxidation. Inhibiting fatty acid transporters or enzymes involved in fatty acid oxidation can impair MDSC function. This approach can potentially reduce MDSC numbers and their immunosuppressive activity, thereby enhancing the antitumor immune response [118,119]. PMN-MDSCs suppress immune responses by increasing the expression of CPT1 and fatty acid uptake to promote fatty acid oxidation in tumors [118]. Overexpression of fatty acid transport protein 2 in PMN-MDSCs contributes to tumor growth through PGE2 synthesis [107]. Additionally, lectin-type oxidized LDL receptor 1, which is highly expressed in PMN-MDSCs, is associated with endoplasmic reticulum stress and lipid metabolism in tumors [120]. Research on therapies targeting lipid metabolism in osteosarcoma highlights this critical aspect of energy metabolism in cancer cells. Osteosarcoma cells utilize lipid metabolism to obtain the energy necessary for tumor growth and metastasis. By regulating lipid metabolism, potential therapeutic strategies to inhibit osteosarcoma growth have been proposed [121]. Additionally, a model that predicts the prognosis of patients with osteosarcoma based on the relationship between lipid metabolism and macrophages has been suggested [122]. Fatty acid amide hydrolase has also been emphasized as an important marker for therapies targeting lipid metabolism in osteosarcoma [123]. Therefore, inhibiting lipid metabolism might be an effective strategy for suppressing cancer cell growth.

### Nanomedicine-based targeting of MDSCs

Cancer treatment strategies using nanoengineering employ nanomaterials to regulate MDSCs in the TME and enhance immune responses. By targeting and inhibiting MDSCs through these techniques, researchers aim to improve the effectiveness of cancer therapies, such as treatment with ICIs or chemotherapy. Targeted delivery using nanoparticles can selectively transport drugs to the TME. These nanoparticles can inhibit the accumulation of MDSCs and block the immunosuppressive signals they release. For example, metal-organic frameworks (MOFs) can be engineered at the nanoscale to deliver therapeutic agents directly to MDSCs, simultaneously promoting tumor suppression and immune activation. IDO, an enzyme expressed by MDSCs, suppresses immune cell activity. Nanoengineering can be used to deliver IDO inhibitors through nanoparticles, preventing MDSCs from releasing immunosuppressive signals and enabling immune cells to attack tumor cells more effectively. Fan et al. developed MOF-based nanomaterials to modulate both IDO and MDSCs, enhancing chemo-immunotherapy for osteosarcoma. By inhibiting the accumulation of MDSCs, the study improved the tumor’s immunosuppressive environment and demonstrated a synergistic effect between chemotherapy and immunotherapy [25]. These approaches aim to reduce or eliminate the immunosuppressive factors in the TME, allowing immune cells to attack the tumor more effectively, thus offering new possibilities for cancer treatment.

### Combination therapies targeting MDSCs

ICIs such as pembrolizumab (anti-PD-1) have shown limited efficacy in some tumors due to the presence of immunosuppressive cells like MDSCs. Combining ICIs with agents that deplete or inhibit MDSCs can enhance the overall antitumor immune responses. Blocking MDSCs can improve the efficacy of ICIs by reducing immunosuppression and promoting effective T-cell functions [94,124]. Combining Adoptive cell transfer (ACT) with MDSC-targeting strategies can improve the persistence and function of the transferred T cells. For example, reducing MDSC levels can decrease the immunosuppressive environment, thus enhancing the efficacy of ACT in eradicating tumors [125]. Although the combination of MDSC inhibition and ACT has the potential to enhance T-cell function and persistence, this has primarily been demonstrated in experimental models, and the clinical efficacy remains uncertain. Combining these strategies with cancer vaccines aims to stimulate the patient’s immune system to recognize and attack tumor cells. Notably, MDSCs can inhibit the efficacy of these vaccines. Combining cancer vaccines with MDSC inhibitors can enhance the vaccine-induced immune response by reducing MDSC-mediated suppression. The study by Iclozan et al., conducted in animal models of small cell lung cancer, showed that combining an MDSC inhibitor with a cancer vaccine enhanced immune responses [126]. Another study highlighted the potential of combining cancer vaccines with ICIs, which can further enhance the antitumor response by reducing MDSC-mediated suppression and blocking other inhibitory pathways used by tumors to evade the immune system [94–96]. Traditional osteosarcoma therapies, such as chemotherapy and radiation therapy, can sometimes paradoxically increase MDSC levels, leading to immune suppression. Combining these therapies with MDSC-targeting agents might mitigate this effect and enhance the overall therapeutic outcome. Gemcitabine and 5-fluorouracil (5-FU) reduce both the number and function of MDSC. These drugs not only diminish tumor growth but also extend survival in mouse models. Clinical observations have further corroborated these findings, demonstrating that gemcitabine and 5-FU decrease MDSC populations and enhance CD8^+^ T-cell responses, which are crucial for effective antitumor immunity [127,128]. Studies in humans and animal models have demonstrated that chemotherapeutic agents, such as platinum-based drugs, cyclophosphamide, gemcitabine, and 5-FU, can reduce MDSC levels, which can help restore the antitumor immune response, making traditional treatments more effective. For example, combining low-dose chemotherapy with fatty acid oxidation inhibitors in animal models delayed tumor growth and reduced MDSC-mediated immunosuppressive activity, thereby enhancing antitumor immunity [129–131]. However, clinical validation remains needed.

Mechanistic insights into the reversibility of epigenetic modifications through small-molecule inhibitors have raised the prospect of targeting specific epigenetic pathways to reprogram the MDSC population into an immunostimulatory phenotype. The histone deacetylase inhibitor entinostat blocks the formation of the pre-metastatic niche by promoting MDSC differentiation into pro-inflammatory macrophages. However, the therapeutic applicability of entinostat in clinical trials has shown limited efficacy [132]. Small-molecule inhibitors, such as STAT3 or CSF1R inhibitors, targeting MDSC pathways have been used in combination with ICIs to improve treatment outcomes. These inhibitors can reduce the number and suppressive function of MDSC, thereby enhancing the antitumor immune response initiated by ICIs [129]. Some studies reported on the combination of MDSC inhibition with ICIs or chemotherapy in osteosarcoma treatment. Inhibition of IL-18-mediated MDSC accumulation improves the efficacy of ICIs, such as anti-PD-1 drugs, in treating osteosarcoma. One study demonstrated a significant reduction in MDSC levels, which enhanced the tumor’s response to immune checkpoint blockade [17]. Another study on tumoral immune infiltrates, PD-L1 expression, and the role of CD8/TIA-1 lymphocytes in patients with localized osteosarcoma examined how MDSCs and ICIs influence tumor progression and how combining these therapies can increase treatment efficacy [133]. The SDF-1–CXCR4 axis facilitates the accumulation of MDCs in the osteosarcoma microenvironment, which blunts the response to anti-PD-1 therapy [22]. Additionally, a study on nanoengineering an MFO for osteosarcoma chemo-immunotherapy shows how modulation of IDO and MDSCs can enhance the effectiveness of chemo-immunotherapy [25]. These studies present promising approaches to improving osteosarcoma treatments by combining MDSC inhibition with ICIs and chemotherapy.

## Future Directions and Conclusion

MDSCs in osteosarcoma have been increasingly recognized as a critical factor in the progression and immune evasion of this aggressive cancer. Thus, targeting MDSCs represents a promising approach to improving the prognosis and survival of patients with osteosarcoma. Elucidating the mechanisms underlying MDSC proliferation and function, as well as their interactions with other immune cells in the TME, is essential for developing effective therapeutic strategies.

However, there are limitations to our review. The preclinical findings need to be validated in clinical settings to assess the efficacy and safety of MDSC-targeting therapies in patients with osteosarcoma. Additionally, while we have focused on the immunosuppressive roles of MDSCs in the TME, further research is necessary to understand the full scope of MDSC interactions with other stromal and cancer-associated cells. Recent studies have demonstrated that MDSCs play dual roles, exerting both immunosuppressive and pro-inflammatory functions. Therefore, the detailed mechanisms and consequences of the pro-inflammatory roles of MDSCs in immune cells in osteosarcoma TME need to be elucidated. In addition, future research should focus on identifying novel targets for modulating MDSC activity and evaluating the efficacy of combination therapies that target multiple components of the TME.

## Data Availability

Not applicable.

## Abbreviations

5-FU: 5-fluorouracil
ACT: Adoptive cell transfer
ARG1: Arginase 1
ATRA: All-trans retinoic acid
C/EBP: CCAAT-enhancer-binding protein
CAR: Chimeric antigen receptor
CCL: C-C motif chemokine ligand
CCR: C-C chemokine receptor
CTL: Cytotoxic T cell
CXCR: C-X-C chemokine receptor
DC: Dendritic cell
G-CSF: Granulocyte colony-stimulating factor
GM-CSF: Granulocyte-macrophage colony-stimulating factor
HK2: Hexokinase 2
ICI: Immune checkpoint inhibitor
IDO: Indoleamine 2,3-dioxygenase
IL: Interleukin
iNOS: Inducible nitric oxide synthase
IRF8: Interferon regulatory factor 8
JAK: Janus kinase 2
M-CSF: Macrophage colony-stimulating factor
M-MDSC: Monocytic myeloid-derived suppressor cell
MCT: Monocarboxylate transporter
MDSC: Myeloid-derived suppressor cell
MOF: Metal-organic framework
NF-κB: Nuclear factor kappa-light-chain-enhancer of activated B cells
NK: Natural killer
NO: Nitric oxide
NOX2: Nicotinamide adenine dinucleotide phosphate hydrogen oxidase 2
PD-1: Programmed cell death protein 1
PD-L1: Programmed death ligand 1
PDE: Phosphodiesterase
PKM2: Pyruvate kinase M2
PMN-MDSC: Polymorphonuclear myeloid-derived suppressor cell
ROS: Reactive oxygen species
STAT3: Signal transducer and activator of the transcription 3
TAM: Tumor-associated macrophage
TCR: T-cell receptor
TME: Tumor microenvironment
VEGF: Vascular endothelial growth factor

## Appendix A

**Table A1:** Therapeutic approaches targeting MDSCs in TME

Type	Name	Mechanism	Status	Reference
Chemical and small molecular drugs	COX-2 inhibitor	COX-2 inhibitor, reduces MDSC function	Preclinical	[134]
	All-trans retinoic acid (ATRA)	Induces MDSC differentiation	Phase I/II	[98]
	Sunitinib	Tyrosine kinase inhibitor, decreases MDSC levels	FDA Approved for RCC	[135]
	Gemcitabine	Chemotherapeutic agent, selectively depletes MDSCs	Phase II/III	[136]
	IDO Inhibitors (Epacadostat)	Inhibits indoleamine-2,3-dioxygenase, modulates MDSC activity	Preclinical	[25]
	CXCR4 Inhibitor (Plerixafor)	Blocks CXCR4/SDF-1 signaling, reducing MDSC recruitment to the tumor microenvironment	Phase I	[22]
	PI3Kδ/γ Inhibitor	Inhibits PI3Kδ/γ, reducing MDSC function and enhancing immune response	Preclinical	[137]
	Arginase Inhibitor	Inhibits arginase activity in MDSCs, restoring T cell function	Preclinical	[138]
	Pexidartinib (PLX3397)	Inhibits the CSF-1/CSF-1R signaling pathway	Phase I/II	[139]
	CXCR2 inhibitor	Decrease the trafficking of MDSCs into TME	Preclinical	[85]
Antibody-based drugs	Anti-PD-1 mAb	Blocks PD-1, counteracting MDSC-induced T cell suppression	Phase I	[17]
	Anti-GR1 mAb	Depletes MDSCs by targeting GR1 antigen	Preclinical	[140]
	Anti-Ly6G mAb	Depletes granulocytic MDSCs	Preclinical	[141]
	Anti-CD33 mAb	Targets MDSCs in myeloid malignancies	Phase I/II	[142]
	Anti-IL-10 mAb	Neutralizes IL-10, reducing MDSC-mediated suppression	Preclinical	[143]
	Anti-EGFR mAb	The M-MDSCs of nonresponders increased and the PMN-MDSCs of responders decreased	Phase II	[144]
	Anti-VEGF mAb	The decrease of PMN-MDSCs	Preclinical	[145]
